# Safety Monitoring of COVID-19 mRNA Vaccine Second Booster Doses Among Adults Aged ≥50 Years — United States, March 29, 2022–July 10, 2022

**DOI:** 10.15585/mmwr.mm7130a4

**Published:** 2022-07-29

**Authors:** Anne M. Hause, James Baggs, Paige Marquez, Winston E. Abara, Jane Baumblatt, Phillip G. Blanc, John R. Su, Brandon Hugueley, Casey Parker, Tanya R. Myers, Julianne Gee, Tom T. Shimabukuro, David K. Shay

**Affiliations:** ^1^CDC COVID-19 Emergency Response Team; ^2^Food and Drug Administration, Silver Spring, Maryland; ^3^Rollins School of Public Health, Emory University, Atlanta, Georgia.

The Advisory Committee on Immunization Practices (ACIP) recommends that all persons aged ≥5 years receive 1 booster dose of a COVID-19 vaccine after completion of their primary series.* On March 29, 2022, the Food and Drug Administration (FDA) authorized a second mRNA booster dose ≥4 months after receipt of a first booster dose for adults aged ≥50 years and persons aged ≥12 years with moderate to severe immunocompromise (*1*,*2*). To characterize the safety of a second mRNA booster dose among persons aged ≥50 years, CDC reviewed adverse events and health impact assessments reported to v-safe and the Vaccine Adverse Event Reporting System (VAERS) after receipt of a second mRNA booster dose during March 29–July 10, 2022. V-safe is a voluntary smartphone-based U.S. active surveillance system that monitors adverse events occurring after COVID-19 vaccination. VAERS is a U.S. passive surveillance system for monitoring adverse events after vaccination, managed by CDC and FDA (*3*). During March 29–July 10, 2022, approximately 16.8 million persons in the United States aged ≥50 years received a fourth dose.^†^ Among 286,380 v-safe registrants aged ≥50 years who reported receiving a second booster of an mRNA vaccine, 86.9% received vaccines from the same manufacturer for all 4 doses (i.e., homologous vaccination). Among registrants who reported homologous vaccination, injection site and systemic reactions were less frequent after the second booster dose than after the first booster dose. VAERS received 8,515 reports of adverse events after second mRNA booster doses among adults aged ≥50 years, including 8,073 (94.8%) nonserious and 442 (5.1%) serious events. CDC recommends that health care providers and patients be advised that local and systemic reactions are expected after a second booster dose, and that serious adverse events are uncommon.

The v-safe platform allows existing registrants to report receipt of a COVID-19 booster dose and new registrants to enter information about all doses received (https://vsafe.cdc.gov/en/). Health surveys sent daily during the first week after administration of each dose include questions about local injection site and systemic reactions and health impacts.^§^ CDC’s v-safe call center contacts registrants who indicate that medical care was sought after vaccination and encourages completion of a VAERS report, if indicated.

VAERS accepts reports of postvaccination adverse events from health care providers, vaccine manufacturers, and members of the public.^¶^ VAERS reports of hospitalization, prolongation of hospitalization, life-threatening illness, permanent disability, congenital anomaly or birth defect, or death are classified as serious.** VAERS staff members assign Medical Dictionary for Regulatory Activities preferred terms (MedDRA PTs) to the signs, symptoms, and diagnostic findings included in VAERS reports.^††^ Reports of serious events to VAERS were reviewed by CDC and FDA physicians to form a consensus clinical impression based on available data. For this analysis, death certificates and autopsy reports were requested for any report of death. CDC physicians reviewed all available information for each decedent to form an impression about the cause of death. For reports of myocarditis and pericarditis, rare adverse events that have been associated with mRNA COVID-19 vaccines, CDC sought information about the clinical course of each case and determined whether the CDC myocarditis case definition was met.^§§^

Local and systemic reactions and health impacts reported during the week after second booster dose vaccination were described for v-safe registrants aged ≥50 years who reported receiving a second booster during March 29–July 10, 2022, ≥4 months after their first booster dose; this analysis was further limited to registrants who received mRNA vaccines for all doses (both homologous and heterologous) and completed at least one daily health survey after receiving their second booster dose and at least one survey after a previous vaccine dose. Among registrants who received homologous mRNA vaccination, the odds of reporting an adverse reaction or health impact after receiving the second booster dose versus previous doses were compared using a multivariable generalized estimating equations model that accounted for demographic variables and repeated measures. Comparisons of adverse reactions and health impacts by vaccine dose were restricted to persons who received homologous mRNA vaccination because previous studies observed different patterns of reporting among recipients of heterologous mRNA and among recipients of homologous Ad26.COV2 (Johnson & Johnson [Janssen]) booster vaccination (*4*). VAERS adverse event reports after a second booster dose were described by serious and nonserious classification, demographic characteristics, and MedDRA PTs. All analyses were conducted using SAS software (version 9.4; SAS Institute); p-values <0.05 were considered statistically significant. These surveillance activities were reviewed by CDC and conducted consistent with applicable federal law and CDC policy.^¶¶^

## Review of v-safe Data

During March 29–July 10, 2022, a total of 286,380 v-safe registrants aged ≥50 years reported receiving a second mRNA vaccine booster dose (homologous or heterologous). The median registrant age was 67 years; 173,525 (60.6%) were female. In the week after receipt of the second booster dose, local injection site reactions were reported by 67,521 (49.1%) BNT162b2 (Pfizer-BioNTech) and 92,472 (62.1%) mRNA-1273 (Moderna) vaccine recipients; systemic reactions were reported by 60,705 (44.2%) Pfizer-BioNTech and 76,756 (51.5%) Moderna vaccine recipients (Table 1). Both local and systemic reactions were mostly mild to moderate in severity and were most frequently reported the day after vaccination. In the week after receipt of the second booster dose, 14,682 (10.7%) Pfizer-BioNTech and 22,385 (15.0%) Moderna vaccine recipients reported inability to complete normal daily activities; 4,300 (3.1%) Pfizer-BioNTech and 5,927 (4.0%) Moderna vaccine recipients reported inability to work or attend school. Receipt of medical care during the week after the second booster vaccination was reported by 0.8% and 0.7% of Pfizer-BioNTech and Moderna vaccine recipients, respectively; most received care via telehealth (0.2% and 0.3%, respectively) or clinic (0.3% and 0.2%, respectively) appointment. Hospitalization was reported by 81 (0.03%) registrants; 39 (48.1%) indicated that the hospitalization was unrelated to vaccination, 28 (34.6%) were unreachable or unwilling to provide additional information, and 14 (17.3%) completed a VAERS report.

**TABLE 1:** Adverse reactions and health impacts reported to v-safe by registrants aged ≥50 years (N = 286,380) who received a COVID-19 mRNA second booster dose,* by vaccine product received — United States, March 29–July 10, 2022

Event	% Reporting reaction or health impact after receipt of second booster dose^†^	
	Pfizer-BioNTech (n = 148,921)	Moderna (n = 137,459)
**Any local injection site reaction**	**49.1**	**62.1**
Itching	5.4	10.2
Pain	45.8	57.2
Redness	5.7	12.4
Swelling	8.9	16.8
**Any systemic reaction**	**44.2**	**51.5**
Abdominal pain	2.4	2.7
Myalgia	20.9	27.2
Chills	9.5	13.8
Diarrhea	4.1	4.3
Fatigue	31.0	37.8
Fever	10.6	15.2
Headache	21.3	26.4
Joint pain	11.8	15.5
Nausea	5.2	6.6
Rash	0.8	1.1
Vomiting	0.5	0.5
**Any health impact**	**12.2**	**16.8**
Unable to perform normal daily activities	10.7	15.0
Unable to work or attend school	3.1	4.0
Needed medical care	0.8	0.7
Telehealth	0.2	0.3
Clinic	0.3	0.2
Emergency visit	0.1	0.1
Hospitalization	0.03	0.03

* Includes only persons who received mRNA COVID-19 vaccine for primary series and first booster dose (homologous and heterologous).

^†^ Percentage of registrants who reported a reaction or health impact at least once during days 0–7 after vaccination.

Among 248,887 (86.9%) v-safe registrants aged ≥50 years who received homologous vaccination and a second mRNA booster dose, local injection site reactions were less frequently reported after the second booster dose than after any previous doses (p<0.001); systemic reactions were less frequently reported after the second booster than after either dose 2 or the first booster dose (p<0.001) (Figure). Inability to complete normal daily activities, to work, or to attend school was less frequently reported after the homologous second booster dose than after either dose 2 or the first booster dose (p<0.001). Receipt of medical care was more frequently reported after the homologous Moderna second booster dose (0.7%) than the first booster dose (0.6%) (p<0.001) and more frequently reported after the second homologous Pfizer-BioNTech dose (0.8%) than the first booster dose (0.6%) (p<0.01).

**FIGURE:**
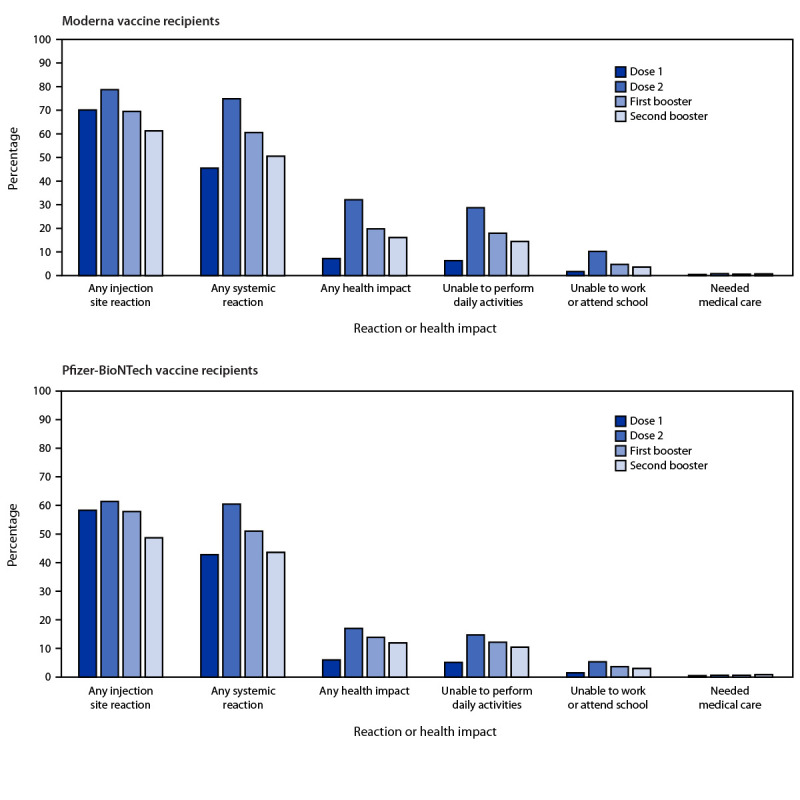
Adverse reactions and health impacts* reported by adults aged ≥50 years who received COVID-19 vaccine booster,^†^ by dose — v-safe data, United States, March 29–July 10, 2022^§^ * Local injection site reactions included itching, pain, redness, and swelling. Systemic reactions included abdominal pain, myalgia, chills, diarrhea, fatigue, fever, headache, joint pain, nausea, rash, and vomiting. Health impacts included inability to perform normal daily activities, inability to work or attend school, and receipt of medical care. ^†^ Adults received either homologous Moderna (125,807) or Pfizer-BioNTech (123,080) COVID-19 vaccine booster doses and completed at least one v-safe health check-in survey on days 0–7 after each vaccine dose. ^§^ The odds of reporting any local injection site or systemic reaction or health impact after second booster dose and previous doses were compared using a multivariable generalized estimating equations model that accounted for the correlation between registrants and adjusted for demographic variables (p-values <0.05 were considered statistically significant); all second booster and first booster dose comparisons were statistically significant.

## Review of VAERS Data

During March 29–July 10, 2022, VAERS received and processed 8,515 reports of one or more adverse events after receipt of a second mRNA booster dose among adults aged ≥50 years (Table 2). The median age of these recipients was 68 years; 5,357 (62.9%) reports were for events among women. Most reports were for nonserious events (8,073; 94.8%), including 2,894 (35.8%) vaccination errors (e.g., expired product administered and product storage error); COVID-19 (2,111; 26.1%); and local and systemic reactions known to be associated with the vaccines and COVID-19, including fatigue (1,236; 15.3%), headache (1,047; 13.0%), and fever (975; 12.1%). Among the 2,894 reports indicating a vaccination error, only 388 (13.4%) also listed an adverse health event, including COVID-19 (74; 19.0%), injection site pain (69; 17.7%), and fever (63; 16.2%) (J. Baggs, PhD, CDC, personal communication, July 2022).

**TABLE 2:** Reports of nonserious and serious events to VAERS among persons aged ≥50 years who received any COVID-19 mRNA second booster dose (N = 8,515) — United States, March 29–July 10, 2022

Reported event	No. (%) reporting
**Nonserious events, VAERS reports**	**8,073 (94.8)**
Symptom, sign, diagnostic result, or condition* (% of total)	
COVID-19	2,111 (26.1)
Expired product administered	1,589 (19.7)
SARS-CoV-2 positive test result	1,443 (17.9)
Fatigue	1,236 (15.3)
Headache	1,047 (13.0)
Fever	975 (12.1)
Cough	911 (11.3)
Pain	810 (10.0)
Product storage error	704 (8.7)
Oropharyngeal pain	655 (8.1)
SARS-CoV-2 test	637 (7.9)
No adverse event^†^	599 (7.4)
Chills	544 (6.7)
Malaise	475 (5.9)
Rhinorrhea	463 (5.7)
**Serious VAERS reports (% of total)**^§,¶,^**	**442 (5.2)^††^**
**Clinical impression (% of serious events)**	
COVID-19	84 (19.0)
Death^§§^	52 (11.8)
Cerebrovascular accident	24 (5.4)
Pulmonary embolism	19 (4.3)
Atrial fibrillation	18 (4.1)
Hearing issue^¶¶^	16 (3.6)
Respiratory infection	9 (2.0)
Hypertension	9 (2.0)
Syncope	9 (2.0)
Fall	8 (1.8)
Transient ischemic attack	8 (1.8)
Arrythmia	7 (1.6)
Myocardial infarction	7 (1.6)
Cognitive concern	6 (1.4)
Thrombosis	4 (0.9)
Coronary artery disease	3 (0.7)
Diabetic ketoacidosis	3 (0.7)
Chronic heart failure	2 (0.5)
Chronic obstructive pulmonary disease	2 (0.5)
Peripheral neuropathy	2 (0.5)
Seizure	2 (0.5)
Shortness of breath	2 (0.5)

**Abbreviations**: MedDRA PT = Medical Dictionary for Regulatory Activities preferred term; VAERS = Vaccine Adverse Event Reporting System.

* Signs and symptoms in VAERS reports are assigned MedDRA PTs by VAERS staff members. Each VAERS report might be assigned at least one MedDRA PT, which can include normal diagnostic findings. A MedDRA PT does not represent a medical diagnosis made or confirmed by a provider or clinical reviewer.

^†^ Reports of no adverse event were accompanied by reports of vaccine error (e.g., expired product administered, product storage error, or extra dose administered).

^§^ VAERS reports are classified as serious if any of the following are reported: hospitalization, prolongation of hospitalization, life-threatening illness, permanent disability, congenital anomaly or birth defect, or death.

^¶^ Serious reports to VAERS were reviewed by CDC physicians to form a clinical impression. The clinical impression of the event does not establish a causal role with vaccination. Reports of myocarditis were identified using a combination of MedDRA PTs; in some cases, reports of myocarditis (identified by fulfilling criteria of the CDC working case definition of myocarditis) did not have the MedDRA PT “myocarditis” assigned to them. https://www.meddra.org/how-to-use/basics/hierarchy

** Cells with fewer than two reports were suppressed.

^††^ Five reports that were duplications were removed from this list.

^§§^ For the six reports of death with sufficient information, cause of death as stated on the death certificate included congestive heart failure, aortic dissection, grand mal seizure, end-stage dementia, and cardiac arrest secondary to coronary artery disease.

^¶¶^ Clinical impressions for “hearing issues” included tinnitus and loss of hearing.

Among the 8,515 VAERS reports of adverse events after receipt of a second mRNA booster dose among adults aged ≥50 years, 12 were preliminary reports of myocarditis (six nonserious and six serious); one case was verified by medical record review and met the CDC case definition for myocarditis; the patient was continuing to recover at time of this report.

Among the 442 (5.2%) reports of serious events after second mRNA booster vaccination among adults aged ≥50 years 52 were reports of death; the median age of decedents was 84 years. For the six reports of death with sufficient information, cause of death as stated on the death certificate included congestive heart failure, aortic dissection, grand mal seizure, end-stage dementia, and cardiac arrest secondary to coronary artery disease. Among the serious events reported, 84 were of COVID-19 (19.0%).

## Discussion

Limited data are available regarding the safety of second COVID-19 booster doses. The findings in this report are consistent with those from a small open-label clinical study of second boosters (154 participants received Pfizer-BioNTech and 120 Moderna vaccines) that did not detect any unexpected safety concerns (*5*). Among 248,887 v-safe registrants aged ≥50 years with homologous vaccination and a second booster dose, injection site and systemic reactions were less frequently reported after the second booster dose than the first booster dose. Similarly, adverse reactions after a first booster dose were less common than were those after dose 2 of the primary series among v-safe registrants aged ≥18 years (*4*) and clinical trial subjects (*6*,*7*). In general, reactions were less frequently reported by v-safe registrants aged ≥50 years after a second booster dose than by adults aged ≥18 years after a first booster dose (*4*); this difference is not unexpected because v-safe participants aged ≥65 years are less likely to report reactions after COVID-19 vaccination than are younger adults (*8*). Overall, 94.8% of VAERS reports were nonserious and vaccine administration errors represented approximately one third of nonserious reports; 13.4% of these also listed an adverse health event. Among both nonserious and serious reports to VAERS, COVID-19 was the most commonly reported event; this is not unexpected given the current epidemiology of the pandemic.

Health care providers are required to report any death that occurs after COVID-19 vaccination to VAERS regardless of whether death has any association with vaccination. Among 40 reports of death after second booster doses, the median decedent age exceeded the median age for all VAERS reports. Reporting rates for death after primary COVID-19 vaccination were higher among adults aged ≥50, consistent with general age-specific mortality rates in the adult population (*9*). After review of available information (six of 40 reports), no vaccine-associated deaths were identified in these reports.

The findings in this report are subject to at least five limitations. First, v-safe is a voluntary reporting program and only 286,380 of the 16.8 million persons aged ≥50 years who received a fourth dose during this period reported receipt to v-safe; therefore, data might not be representative of the entire vaccinated U.S. population. Second, vaccine recipients who experience an adverse event could be more likely to respond to v-safe surveys. Third, VAERS is a passive system and is subject to reporting biases and underreporting, especially of nonserious events (*3*). Fourth, medical review of reported deaths after vaccination is dependent on availability of medical records, death certificates, and autopsy reports, which might be unavailable or not available in a timely manner. Finally, a report to v-safe or VAERS alone cannot be used to assess causality.

ACIP recommends that all persons aged ≥5 years receive one booster dose of a COVID-19 vaccine after completion of their primary series; adults aged ≥50 years and persons aged ≥12 years with moderate to severe immunocompromise might receive a second booster ≥4 months after their first booster dose. Among adults aged ≥50 years, vaccine effectiveness against COVID-19-associated hospitalization ≥120 days after dose 3 was 55% and ≥7 days after dose 4 was 80%, reinforcing recommendations that persons in this age group should receive a second booster when they become eligible (*10*). Preliminary safety findings after receipt of second booster doses among adults aged ≥50 years are similar to those after receipt of first booster doses. Reports of reactions after the second booster dose are less common than are those after the first. Health care providers and patients should be advised that local and systemic reactions are expected after second booster doses and that serious adverse events are infrequently reported. CDC and FDA will continue to monitor vaccine safety and will provide updates as needed to guide COVID-19 vaccination recommendations.

**Summary**

**What is already known about this topic?**

During March 29–July 10, 2022, approximately 16.8 million persons in the United States aged ≥50 years received a fourth dose of a COVID-19 vaccine.

**What is added by this report?**

Among persons aged ≥50 years who reported homologous mRNA COVID-19 vaccination, injection site and systemic reactions were less frequent after a second booster dose than after the first booster dose. Ninety-five percent of 8,515 events reported to the Vaccine Adverse Event Reporting System were nonserious.

**What are the implications for public health practice?**

Health care providers and patients should be aware that local and systemic reactions are expected after a second mRNA COVID-19 booster dose. Serious adverse events are uncommon.

## Footnotes

* ACIP recommends that all persons aged ≥5 years receive 1 booster dose of a COVID-19 vaccine after completion of their primary series (≥5 months after BNT162b2 [Pfizer-BioNTech] or mRNA-1273 [Moderna] primary series and ≥2 months after Ad26.COV2 (Johnson & Johnson [Janssen] primary series); mRNA vaccine is preferred over Janssen for the first booster dose. https://www.cdc.gov/vaccines/covid-19/clinical-considerations/covid-19-vaccines-us.html (Accessed July 12, 2022).

† https://covid.cdc.gov/covid-data-tracker/#cases_casesper100klast7days (Accessed July 12, 2022).

§ Health surveys were sent for the most recent dose entered via text messages that link to web-based surveys on days 0–7 after receipt of a vaccine dose; then weekly through 6 weeks after vaccination; and then at 3, 6, and 12 months after vaccination. Local injection site reactions included itching, pain, redness, and swelling. Systemic reactions included abdominal pain, myalgia, chills, diarrhea, fatigue, fever, headache, joint pain, nausea, rash, and vomiting. Health impacts included inability to perform normal daily activities, inability to work or attend school, and receipt of medical care.

¶ Health care providers are encouraged by CDC and FDA to report adverse events to VAERS and are required by COVID-19 vaccine Emergency Use Authorizations to report certain adverse events after vaccination to VAERS, including death (https://vaers.hhs.gov/faq.html). A VAERS form includes patient information, vaccine information, vaccine administration information, and information regarding the adverse event (https://vaers.hhs.gov/docs/VAERS%202.0_Checklist.pdf).

** VAERS reports are classified as serious based on the Code of Federal Regulations Title 21. https://www.accessdata.fda.gov/scripts/cdrh/cfdocs/cfcfr/cfrsearch.cfm?fr

†† Each VAERS report might be assigned at least one MedDRA PT. A MedDRA coded event does not indicate a medically confirmed diagnosis. https://www.meddra.org/how-to-use/basics/hierarchy

§§ Reports of myocarditis and pericarditis were identified in VAERS by searching for selected MedDRA PTs. In VAERS, acute myocarditis was defined as presence of signs and symptoms including new onset or worsening of at least one of the following signs or symptoms: chest pain, pressure, discomfort, dyspnea, shortness of breath, pain with breathing, palpitations, or syncope; or at least two of the following signs or symptoms in children aged ≤11 years: irritability, vomiting, poor feeding, tachypnea, or lethargy; and at least one new finding of elevated troponin, electrocardiogram findings consistent with myocarditis, abnormal cardiac function or wall motion on echocardiogram, cardiac magnetic resonance imaging findings consistent with myocarditis, or histopathologic findings consistent with myocarditis; and no other identifiable cause for these findings.

¶¶ 45 C.F.R. part 46, 21 C.F.R. part 56; 42 U.S.C. Sect. 241(d); 5 U.S.C. Sect. 552a; 44 U.S.C. Sect. 3501 et seq.
